# Modulating protein quality control

**DOI:** 10.7554/eLife.18431

**Published:** 2016-07-20

**Authors:** Lars Plate, Ryan J Paxman, R Luke Wiseman, Jeffery W Kelly

**Affiliations:** 1 Department of Chemistry, The Scripps Research Institute, La Jolla, United States; 1 Department of Chemistry, The Scripps Research Institute, La Jolla, United States; 1 Department of Chemistry, The Scripps Research Institute, La Jolla, United Statesjkelly@scripps.edu; 2 Department of Molecular and Experimental Medicine, The Scripps Research Institute, La Jolla, United States; 2 Department of Molecular and Experimental Medicine, The Scripps Research Institute, La Jolla, United Stateswiseman@scripps.edu; 2 Department of Molecular and Experimental Medicine, The Scripps Research Institute, La Jolla, United States; 3 Department of Chemical Physiology, The Scripps Research Institute, La Jolla, United States

**Keywords:** endoplasmic reticulum, unfolded protein response, small molecule screening, ATF6-alpha, ER stress, site-1-protease, Human

## Abstract

Small molecules that modulate the unfolded protein response have the potential to treat a variety of human protein misfolding diseases.

**Related research articles** Gallagher C, Garri C, Cain E, Ang K, Wilson C, Chen S, Hearn B, Jaishankar P, Andrada-Diaz A, Arkin M, Renslo A, Walter P. 2016. Ceapins are a new class of unfolded protein response inhibitors, selectively targeting the ATF6α branch. *eLife* **5**:e11878. doi: 10.7554/eLife.11878

Gallagher CM, Walter P. 2016. Ceapins inhibit ATF6α signaling by selectively preventing transport of ATF6α to the Golgi apparatus during ER stress. *eLife* **5**:e11880. doi: 10.7554/eLife.11880

Plate L, Cooley C, Chen J, Paxman R, Gallagher C, Madoux F, Genereux J, Dobbs W, Garza D, Spicer T, Scampavia L, Brown S, Rosen H, Powers ET, Walter P, Hodder P, Wiseman RL, Kelly JW. 2016. Small molecule proteostasis regulators that reprogram the ER to reduce extracellular protein aggregation. *eLife* **5**:e15550. doi: 10.7554/eLife.15550

**Image** Ceapins make cells more sensitive to endoplasmic reticulum stress

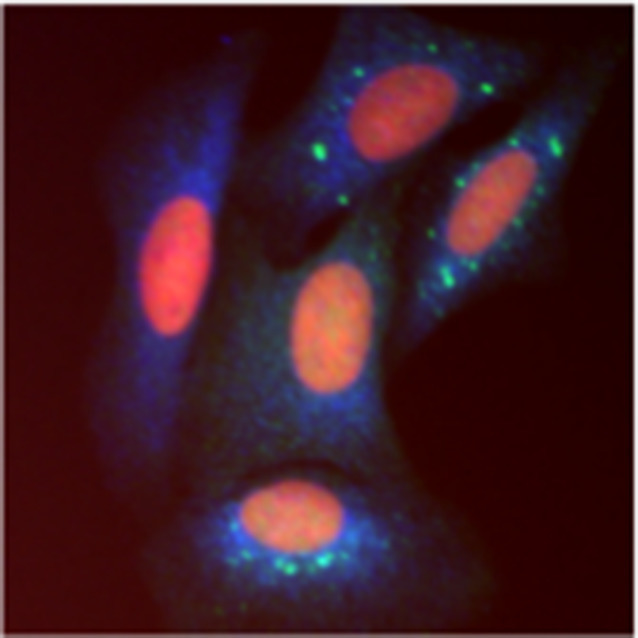

A third of all human proteins are folded inside a compartment called the endoplasmic reticulum with the help of chaperone proteins (Balch et al., 2008). This compartment also contains other protein-based factors that check the quality of the folded proteins and send them to be degraded if they do not meet the required standard. If unfolded or misfolded proteins start to accumulate, the endoplasmic reticulum becomes stressed and activates a signaling pathway called the unfolded protein response (Mori, 2000; Kaufman, 2002; Ron and Walter, 2007). There are three stress-sensor proteins that each control different branches of the unfolded protein response. ATF6 and IRE1 lead to the upregulation of genes that enhance the capacity of the endoplasmic reticulum to fold proteins or mediate quality control, whereas PERK performs several other roles including pausing the production of new proteins to temporarily lower the protein-folding burden.

The unfolded protein response is implicated in many diseases. For example, the response is often activated in rapidly growing cancer cells, which enables the cells to fold the large numbers of mutated proteins they produce. Also, viruses can trigger this response as part of their strategy to trick host cells into producing a number of difficult to fold viral proteins. Thus, inhibiting this stress-responsive signaling pathway is a promising way to treat cancer and viral infections (Tardif et al., 2002; Ma and Hendershot, 2004). In contrast, other diseases are linked to insufficient unfolded protein response signaling. For example, human amyloid diseases are caused by certain proteins that are prone to misfolding escaping endoplasmic reticulum quality control and forming toxic clumps outside cells (Shoulders et al., 2013). The ability to activate one or more branches of the unfolded protein response may make it possible to develop new therapies for these diseases.

Previous studies have identified several small molecules that activate or inhibit the IRE1 or PERK branches of the unfolded protein response, and these molecules have shown promise for influencing diverse human diseases (Papa et al., 2003; Kudo et al., 2008; Wiseman et al., 2010; Tsaytler et al., 2011; Wang et al., 2012; Sidrauski et al., 2015; Robblee et al., 2016). However, very few small molecules that modulate the ATF6 branch have been found, partly because little was known about how this branch is activated.

It is well established that endoplasmic reticulum stress induces the transport of full-length ATF6 to the Golgi, where it is cut into fragments by two protease enzymes. The cytosolic fragment containing the active transcription factor domain of ATF6 then moves to the nucleus and alters gene expression (Figure 1). The identification of small molecules that influence ATF6 activation has also been hampered by three factors: the lack of sites on ATF6 that are known to be able to bind to small molecules; the lack of structural information about the protein; and the fact that ATF6 is not readily amenable to biochemical high-throughput screening approaches (it is a transmembrane protein).

**Figure 1.**
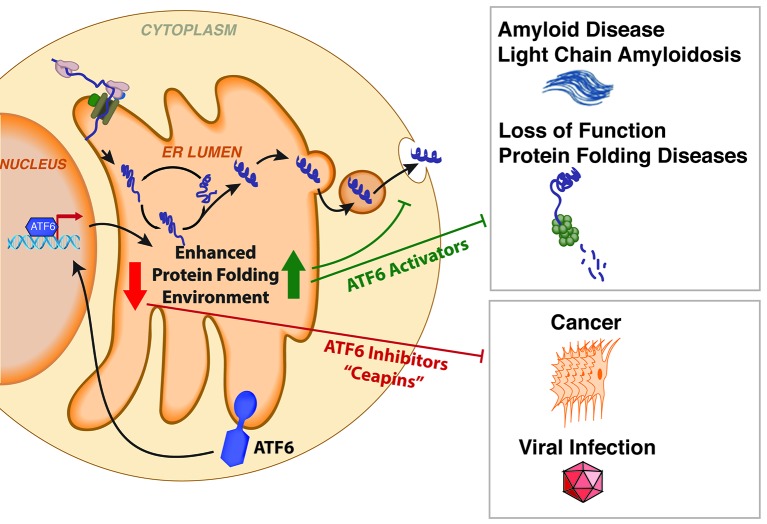
Small molecules modulate protein folding in the endoplasmic reticulum and influence disease. Many proteins are folded inside a compartment called the endoplasmic reticulum (ER) before being secreted from the cell. Misfolded proteins in the endoplasmic reticulum activate the stress-sensor protein ATF6, which upregulates genes that increase the capacity of the endoplasmic reticulum to fold proteins. ATF6 signaling is often active for extended periods of time in cancer cells and virus-infected cells, which enables these cells to fold large numbers of proteins needed for the cancer or virus to spread. The Ceapin molecules reported by Gallagher et al. can inhibit this chronic ATF6 signaling, and make these cells more sensitive to endoplasmic reticulum stress. In our study, we identified a set of small molecules that activate ATF6 signaling and increase the capacity of the endoplasmic reticulum to fold proteins in the absence of stress. These molecules can reduce the secretion of misfolded proteins that are associated with amyloid diseases, and possibly prevent other protein folding diseases.

Now, in two papers in eLife, Peter Walter of the University of California, San Francisco and co-workers used a cell-based screen to identify a class of pyrazole amides called the Ceapins as inhibitors of ATF6 stress-responsive signaling (Gallagher et al., 2016; Gallagher and Walter, 2016). And in related work, the present authors, in collaboration with Walter and colleagues, identified a set of small molecules that activate ATF6 signaling (Plate et al., 2016).

Walter and co-workers – including Ciara Gallagher as first author – found that Ceapins specifically block ATF6 signaling in response to endoplasmic reticulum stress, while not affecting signaling through the IRE1 and PERK branches of the unfolded protein response (Gallagher et al., 2016). Ceapins act upstream of the protease enzymes that cut ATF6, and do not affect signaling from two similar proteins that are also targeted by these enzymes.

In an accompanying paper, Gallagher and Walter used biochemical and cell biology approaches to demonstrate that Ceapins trap ATF6 in particular spots in the endoplasmic reticulum that are away from the places where proteins leave this compartment (Gallagher and Walter, 2016). This arrangement prevents ATF6 from being moved to the Golgi upon stress, thus preventing the release of the transcription factor domain. Notably, Ceapins sensitize cells to endoplasmic reticulum stress without affecting the ability of unstressed cells to survive. This indicates that Ceapins could be used alone or in combination with other drugs, such as bortezomib, to induce cell death in certain types of cancer.

In our study, we employed a different cell-based screening approach to identify a collection of non-toxic small molecules that preferentially activate the ATF6 branch of the unfolded protein response, while minimizing activation of the IRE1 and PERK branches (Plate et al., 2016). The ability of these small molecules to activate this branch requires both endogenous ATF6 and protease activity. Collectively, our results confirm that these molecules activate ATF6 through its normal mechanism in the absence of endoplasmic reticulum stress. Moreover, these molecules enhance quality control in the endoplasmic reticulum to selectively reduce the secretion and extracellular aggregation of the proteins responsible for amyloid diseases. Therefore, these molecules could become promising drug leads for treating amyloid diseases, and potentially other protein misfolding diseases.

The discovery of the Ceapins and ATF6 activator compounds – along with the previously known activators and inhibitors of the PERK and IRE1 pathways – now enables all three branches of the unfolded protein response to be modulated, at least in isolated cells. This chemical toolbox offers the unique opportunity to define the therapeutic potential of altering unfolded protein response signaling in a variety of disease models. Future medicinal chemistry and pharmacology studies have the potential to provide new drugs that work by altering ATF6 signaling.
